# Surface electromyography as a screening method for evaluation of dysphagia and odynophagia

**DOI:** 10.1186/1746-160X-5-9

**Published:** 2009-02-20

**Authors:** Michael Vaiman, Ephraim Eviatar

**Affiliations:** 1 Department of Otolaryngology, Assaf Harofe Medical Center, Affiliated to Sackler Faculty of Medicine, Tel Aviv University, Israel

## Abstract

**Objective:**

Patients suspected of having swallowing disorders, could highly benefit from simple diagnostic screening before being referred to specialist evaluations. The article analyzes various instrumental methods of dysphagia assessment, introduces surface electromyography (sEMG) to carry out rapid assessment of such patients, and debates proposed suggestions for sEMG screening protocol in order to identify abnormal deglutition.

**Data sources:**

Subject related books and articles from 1813 to 2007 were obtained through library search, MEDLINE (1949–2007) and EMBASE (1975–2007).

**Methods:**

Specifics steps for establishing the protocol for applying the technique for screening purposes (e.g., evaluation of specific muscles), the requirements for diagnostic sEMG equipment, the sEMG technique itself, and defining the tests suitable for assessing deglutition (e.g., saliva, normal, and excessive swallows and uninterrupted drinking of water) are presented in detail. SEMG is compared with other techniques in terms of cost, timing, involvement of radiation, etc.

**Results:**

According to the published data, SEMG of swallowing is a simple and reliable method for screening and preliminary differentiation among dysphagia and odynophagia of various origins. This noninvasive radiation-free examination has a low level of discomfort, and is simple, time-saving and inexpensive to perform. The major weakness of the method seems to be inability for precise diagnostic of neurologically induced dysphagia.

**Conclusion:**

With standardization of the technique and an established normative database, sEMG might serve as a reliable screening method for optimal patient management but cannot serve for proper investigation of neurogenic dysphagia.

## Introduction

Swallowing disorders comprise an interdisciplinary phenomenon. Practitioners in various fields of medicine, such as otorhinolaryngology, neurology, general medicine, gastroenterology, head and neck surgery, dentistry and facial surgery, pediatrics and psychiatry deal with these disorders regularly, but family doctors and emergency department personnel might well be the first physicians to evaluate these patients.

### Basic terminology

Dysphagia is a difficult or abnormal swallowing, which can include nasopharyngeal regurgitation and aspiration [1]. It is also defined as any defect in the intake or transport of saliva, liquids and food necessary for the maintenance of life. Odynophagia is a painful swallowing. Odynophagia is not a constant pain in the throat but rather pain on swallowing (swallow-evoked pain) [2]. It was F. Magendie who, in 1813, established the concept of three stages in the act of swallowing: oral, pharyngeal, and oesophageal [3,4]. Since the 1980s, the act of swallowing has sometimes been described as consisting of four stages, with the oral stage being divided into oral initial (for solids – oral preparation stage) and oral final stages [5]. In case of liquid swallowing, during the oral initial stage the water intake takes place and a labial seal occurs. The oral final stage occurs when the tongue squeezes the liquid volume against the hard palate so that it is propelled past the anterior faucial arches. At this stage the automatic reflexive gesture of swallowing is triggered, but the oral stage itself remains under complete conscious control. During the pharyngeal stage the liquid volume is transferred from the level of the faucial arches through the pharynx to the cricopharyngeal sphincter at the rostral aspect of the esophagus. This stage is often described as a reflex, i.e. a person cannot stop swallowing in the middle of the process. In the oesophageal stage of the swallow, the water volume is transferred in a continuation of the peristaltic movement from the cricopharyngeal to the gastro-oesophageal sphincter at the entrance to the stomach.

## Background

The above described combination of voluntary and involuntary stages in deglutition makes evaluation of swallowing pathophysiology a difficult and sometimes timely and expensive process. Different diagnostic techniques were proposed for it. 20 years ago, in 1988, in a general description of evaluation of swallowing pathophysiology, the list of these techniques was as follows: still X-ray, computerized axial tomography (CAT), magnetic resonance imaging (MRI), indirect laryngoscopy, pharyngeal manometry, scintigraphy, ultrasound, videofluoroscopy [6]. Electromyography (EMG) was not in this list. 10 years ago, in 1998, the list was somewhat changed and included barium esophagram, air contrast esophagram, manometry, manofluorography, flexible endscopic evaluation of swallowing with sensory testing (FEESST), bolus scintigraphy, ultrasonography, videofluoroscopic swallowing study (VFSS), and videoendoscopic swallowing study (VESS) [7]. Once again, EMG was not mentioned.

At that moment, disadvantages for each of the above mentioned methods were well known. In brief, they are:

barium esophagram – uses x-irradiation, needs a facility and personnel, no dynamic futures;

air contrast esophagram – the same as above [8,9];

manometry – cannot diagnose visible lesions, unpleasant, can be unvalid because of movement of larynx [10,11];

manofluorography – not widely available, costly [12,13];

FEESST – requires complicated equipment, not widespread, expensive [14,15];

bolus scintigraphy – cannot see patient anatomy, uses isotopes [7];

ultrasonography – segmental, cannot present panoramic anatomic detail, expensive [7];

VESS – the phases of swallowing cannot be seen directly, and also aspiration that occurs during the swallow, the function of the cricopharyngeus muscle cannot be directly assessed [16].

VFSS – relies on x-irradiation, needs radiology equipment and personnel, expensive [7];

Currently VFSS is the most commonly used tool in the assessment of oropharyngeal dysphagia, and it is considered the gold standard in the dysphagia workup. But in addition to above mentioned drawbacks, VFSS does not always identify neuromuscular abnormalities in pharyngeal or laryngeal physiology.

In addition to the above, one can see that all these methods hardly can be used for rapid screening purposes. Comparison between swallowing studies options in respect to radiation, timing and cost is shown in the Table 1. While cost of the procedures varies from country to country, simplified "inexpensive – moderate – expensive" scale is used. Total time needed per person per procedure is a combination of the time needed for preparation of a patient, time needed for preparation of an instrument, actual time of a procedure, and the time needed for data interpretation and report. The timing also depends of how well the personnel are trained.

**Table 1:** Comparison between swallowing studies options in respect to radiation, timing and cost

**Procedure**	**Radiation**	**Time**	**Cost**
Barium Esophagram	Yes	Moderate	Moderate

Air Contrast Esophagram	Yes	Moderate	Moderate

Manometry	No	Time-consuming	Inexpensive

Manofluorography	Yes	Time-consuming	Expensive

FEESST	No	Time-consuming	Expensive

Bolus Scintigraphy	Yes	Moderate	Moderate

Ultrasonography	No	Moderate	Expensive

VESS	No	Time-consuming	Inexpensive

VFSS	Yes	Time-consuming	Expensive

Surface EMG	No	Time-saving	Inexpensive

* Goes in two stages, the examination is videotaped and then again analyzed.

** in addition to radiologist, speech pathologists must be present.

### Do we need dysphagia screening?

Dysphagia occurs in approximately 14% of patients in acute care setting an up to 50% of patients in nursing homes [17]. Its prevalence is related to the fact that dysphagia often is present in patients who have sudden-onset ENT or neurologic disorders [18], head and neck cancers, chronic neurodegenerative diseases, and patients with general medical problems, but in general it is indeed an interdisciplinary phenomenon. Even practitioners in the field of pediatrics [19,20] deal with these disorders. However, more than 75% of cases of oropharyngeal dysphagia are caused by ENT problems or neuromuscular disorders [21]. To carry out the rapid assessment of patients with dysphagia and/or odynophagia requires a simple screening diagnostic tool to be used before extensive clinical and instrumental examination will be performed by a specialist. The diagnostic tool should be

• reliable

• preferably noninvasive

• preferably radiation-free

• inexpensive

• time saving

• providing both qualitative and quantitative data

• simple and easy to operate

In general, aim of a screening procedure is to separate patients into a "passed"- vs. "failed" group, specifically into groups with normal and abnormal deglutition. Screening so far is a filter to decide whether further clinical diagnostics or treatment is necessary. In case of dysphagia, however, the second step after normal-abnormal screening is needed. This step might answer the question to what specialist a dysphagic patient should be referred to: neurologist? ENT-physician? dentist? gastroenterologist? psychiatrist? This second screening stage is actually more practicable comparing with the first passed-failed one because initial dysphagia detection starts with a history taking.

Swallowing is a muscular process. Its mechanism, by which food is transmitted to the stomach, is a complex action involving 26 muscles and five cranial nerves [22]. This fact suggests that surface electromyography (SEMG) might be a valuable method to be used for screening purposes and early diagnostics of dysphagia and odynophagia complaints. Indeed, surface SEMG provides information on the timing of selected muscle contraction patterns during swallowing [23-25], amplitude of electric activity of the muscles [26], and can be easily learned by the personnel [27,28]. Some suggestions have been already made for using SEMG for screening purposes in neurogenic dysphagia [29].

Despite numerous studies of SEMG activity of face and neck muscles during swallowing made in the 1990s [23-30], lack of agreement arose both in some of the basic aspects common to all subjects and in establishing normal limits of this activity beyond which the act becomes pathological. That is why the first step was to establish a normative database for the phenomenon, and it was reported in numerous studies [31-36]. A review of 440 healthy adults (230F, 210M, age mean 31.8 years) in one study and 300 adults (170F, 130M, age mean 33.9 years) in another study provided data which we used as a basis for comparison of swallowing performance both within and between patients. Quick reference simplified set of normative data for electric activity obtained by surface EMG for masseter (MS) and submental muscle group + platisma (SUB) during various tests is presented in the Table 2[33].

**Table 2:** Quick reference simplified set of normative data for electric activity obtained by surface EMG for masseter and submental group + platisma during various tests, in μV

**Saliva swallow**			
masseter range	18–30: **4.5 – 15.9**	31–70: **5.54 – 12.1**	70+: **2.94–22.42**

Submental range	18–30: **13.4–59.72**	31–70: **9.52 – 49.5**	70+: **10.2–42.32**

**Normal swallow**			

masseter range	18–60:**2.2–31.0**	61–70: **1.97 – 27.69**	70+: **3.77–20.0**

submental range	18–30:**11.4–63.41**	31–50: **12.58–51.6**	51–70+: **7.4 – 44.8**

**20 cc excessive swallow**			

masseter range	18–40:**1.5–37.0**	41–70:**1.2 – 29.4**	70+: **4.65–21.13**

submental range	18–30:**19.28–50.80**	31–70+: **12.1 – 47.44**	

**100 cc drinking**			

masseter mean (real)	18–70: **0.8 – 6.2**	70+: **1.0 – 7.84**	

submental mean (real)	18–60: **3.5 – 11.5**	61 – 70+: **4.25 – 16.25**	

[age: **normal values**]

Up to date, however, we face various technical approaches to SEMG usage in evaluation of swallowing process. While the normative database for SEMG assessment of deglutition has been established, the next step might be establishment of standards for this diagnostic procedure, i.e. the protocol. Large variation in examination techniques, strategies, interpretations and diagnostic criteria have been found among electromyographers and it is suggested that the value of SEMG studies of deglutition may be further improved by international standardization.

### Surface EMG as a screening method

SEMG is to be clearly separated from needle electrode examination EMG [37]. Needle electrodes should be used within the neck area with great precaution and this procedure will be neither time-saving, nor noninvasive. The needle EMG report includes tabulated nerve conduction studies, which are not needed at the screening stage of investigation.

Reliability of the method was proved in a series of research using SEMG for diagnostic purposes in oral [38,39], pharyngeal [40-43] and esophageal [44] diseases. These investigations suggested that different diseases have specific SEMG patterns, both in timing and amplitude of the record. The graphic record itself has visible peculiarities specific for each disease. Filtered SEMG provides a simple EKG-looking line easy to analyze and interpret.

If a diagnostic method is offered to be used in different areas of medicine but for one purpose, the standard protocol is a must. The need for established protocols in SEMG investigations is well understood [45-48], and several attempts already have been made [49,50]. To our knowledge, no such protocols were proposed for SEMG evaluation of dysphagia and odynophagia. SEMG evaluation of deglutition is not a new diagnostic method. Nevertheless, lack of standard requirements decrease the outcome of this investigation technique significantly. In the case of SEMG evaluation of deglutition the protocol might be based on:

• protocol application

• protocol requirements for diagnostic equipment

• protocol technique

• protocol tests

• normative database and standard analysis

### Suggestions for protocol application

As it was mentioned above, the act of swallowing is described as consisting of four stages, as the oral stage was divided into oral initial (for solids – oral preparation stage) and oral final stages [5]. This staging can be helpful in diagnostic evaluation of disorders providing dysphagia and odynophagia. Each of these stages can be impaired and the screening evaluation should be capable to indicate the impaired stage. Surface SEMG recording cannot trace oesophageal activity and only initial oesophageal stage can be recorded and evaluated. SEMG does not detect silent breathing.

### Protocol equipment

**Figure 1:**
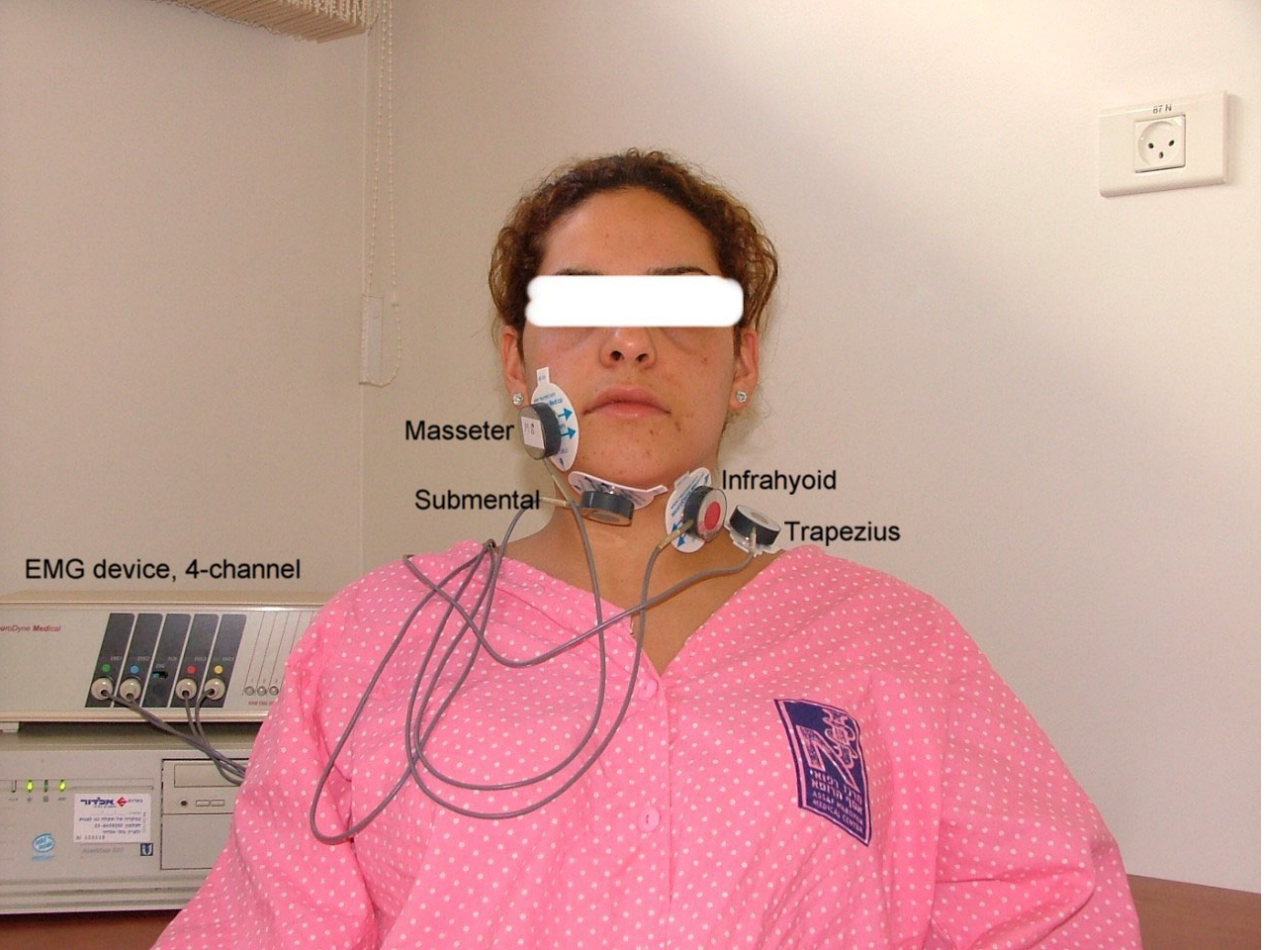
**The electroneuromyograph at work**. A subject with masseter, submental group, infrahyoid, and trapezius locations of SEMG electrodes.

Surface SEMG devices are non-invasive, radiation-free, generally inexpensive and as simple as standard EKG equipment [51]. Four-channel computer-based SEMG unit equipped with surface electrodes is usually enough. (Fig. 1). Two-channel unit is inadequate and eight-channel unit being useful in scientific research is not convenient for rapid screening procedure. Standard surface electrodes AE-131 and AE-178 are sufficient. (Electrode material: silver coated; shape: discs; size: diameter 11 mm; the inter-electrode distance: 10 mm). However other surface electrodes with similar characteristics can be applied [52]. SEMG recordings are to be preferably performed by a unit which has a wide bandpass filter, a bandwidth (RMS) of 25–450 Hz and a 60 Hz notch filter. The system uses an active electrode consisting of a compact sensor assembly that includes a miniaturized instrument preamplifier. Locating the amplifier at the electrode site allows cancellation of artifacts and boosting of the signal before it is transferred down the electrode cable. The integration period of the sSEMG signal at the hardware level is 25 milliseconds. This has very little effect on the shape of the signal [53]. For the software, the underlying sampling speed is 100 Hz (i.e., 100 samples per second). The recordings of this high-speed sampling are then averaged, based on the selected sampling rate. Each sSEMG recording should be full-wave rectified and low-passed filtered (the remote preamplifier, low-pass cutoff = 30 Hz). The computer program should be able at least to calculate the mean, standard deviation, minimum, maximum, and range of muscle activity during each trial, as well as its duration. Muscle activity (SEMG) is quantified in microvolts (μV).

Any other SEMG device with similar characteristics can be used but one quality has to be insured: the SEMG record has to be full-wave rectified and low-passed filtered in a way to look like a single EKG-looking line. SEMG records with numerous closely packed spikes are almost impossible to interpret rapidly. If the four-channel device is used, the investigation can be performed in 3–5 minutes in cases when we have full cooperation of a patient.

### Electromyographic technique

Four muscle groups are to be examined during the evaluation [31-36]:

(1) for oral phase: the orbicularis oris superior and inferior (OO),

(2) for oral phase: the masseter (MS),

(3) for pharyngeal phase: the submental muscle group (SUB) which includes the anterior belly of the digastric, mylohyoid, and geniohyoid, all covered by the platysma,

(4) for pharyngeal and initial oesophageal phases: (INF), the infrahyoid group, thyrohyoid, and the laryngeal strap muscles also covered by the platysma.

Protocol electrode positions might be as follows [31-36]:

(1) Two bipolar stick-on surface electrodes are to be applied at the right or left angle of mouth, one electrode above the upper lip, and another electrode below the lower lip (OO-location);

(2) Two electrodes are to be placed parallel to the masseter muscle fibers on the left or right side of the face, preferably on the opposite side from the OO-location (MS-location);

(3) Two surface electrodes are to be attached to the skin beneath the chin on the right or left side of midline to record SUB myoelectrical activity over the platysma (SUB-location);

(4) Two electrodes are to be placed on the left or right side of the thyroid cartilage to record from the laryngeal strap and infrahyoid muscles (INF-location).

#### Variation

when OO-location is uninformative, but some general muscular-neurologic disease is suspected, the fourth electrode can be attached above a muscle not involved in deglutition, for example, above m. trapezius (Fig. 1). The same variation is preferable in cases of psychogenic swallow or malingering, when a patient strains muscles not involved in deglutition.

The exact electrode positions for each muscle group have been known since the 19^th ^century [54,55], and, in addition, can be clarified following anatomical correlates [56]. The proposed electrode locations cover all stages of a swallow. The staging of normal deglutition can be clinically important as an additional tool for establishing aetiology and localization – oral, pharyngeal, or oesophageal – of causes for dysphagia or odynophagia. Each stage has its mean normal duration and its specific graphic pattern. Each pair of electrodes has a third electrode as ground. Electrical impedance at the sites of electrode contact might be reduced since the target areas are lightly scrubbed with alcohol gauze pads. Radiates skin, edematous skin and status post neck dissection are possible counter-indications for SEMG investigation.

### Protocol tests

A set of four tests might be suggested: voluntary single swallows of saliva ("dry" swallow), voluntary single water swallows from an open cup ("normal"), voluntary single swallows of an excessive amount of water (20 ml, "stress test"), and continuous drinking of 100 cc of tap water from an open cup [31-36,38-44]. Subjects are permitted to move their chins slightly upwards while swallowing if needed when it emerged that there is no changes of the graphic and numerical baseline associated with this movement. (This movement involves the mm. rectus capitis posterior minor and minor, as well as some other posterior neck muscles, and does not affect signals from the above-mentioned electrode locations.) After electrode placement, a patient is asked to perform the following tasks:

1. Three trials of "dry" swallowing. Instruction given: "Swallow your saliva".

2. Three trials of swallowing normal volume of tap water for a particular person. These volumes, calculated for a particular age group [31-36], are: Group 1 (age 18–40) – 16,5 cc; Group 2 (age 41–70) – 14,5 cc; Group 3 (age 70+) – 12 cc. Instruction given: "Swallow this water in one gulp".

3. Three trials of swallowing 20 cc of tap water to check adaptation abilities of the patients ("stress test", larger bolus volume accommodation). Instructions given: "Swallow this water in one gulp".

4. One trial of continuous drinking of 100 ml of tap water. Instruction given: "Drink this water as normal".

The main test is a single water swallow as normal. Saliva swallow might be very important test in case of salivary glands diseases like, for example, Sjögren syndrome. Stress test with excessive amount of water swallowed in one gulp might reveal lack of larger bolus volume accommodation abilities in cases of anatomical changes of the pharynx, neurological problems or might provoke regurgitation in patients with Zenker's diverticulum. Testing of continuous drinking is important not only in the evaluation of dysphagia but also of odynophagia and in the differential diagnosis in cases of compulsive water drinking, excessive water drinking, the malingering of dysphagia and psychogenic disorders expressed by symptoms of dysphagia. This is the test that can help in better evaluation of slight dysphagia in fast-fatigable subjects. For fast-fatigable subjects continuous non-interrupted drinking is a stress test [57]. The amount of water for continuous drinking test was set at 100 cc, approximately one-half of a standard glass because a smaller volume, e.g., 50 cc, can be swallowed in two gulps and provide inadequate data while we had observed that 200 cc of water involves significant swallowing/ventilation interactions which can confound the validity of the obtained data.

In addition to water swallow tests, there are tests with food, involving mastication. They can be justified in cases of diseases of temporomandibular joint and some other disorders. For screening purposes, however, simple water swallow tests are sufficient.

### Protocol analysis

Protocol analysis includes assessment of duration (in sec), amplitude of electric activity (mean, in μV), graphic patterns and number of swallows (in continuous drinking). To carry out the rapid assessment of patients, the SEMG record should be clear and easily understandable. A comprehensive study was recently performed in which SEMG was used for monitoring functionally distinct muscle activation during swallowing [58]. This study supports the statement that raw SEMG records should be rectified and filtered before evaluation.

**Figure 2:**
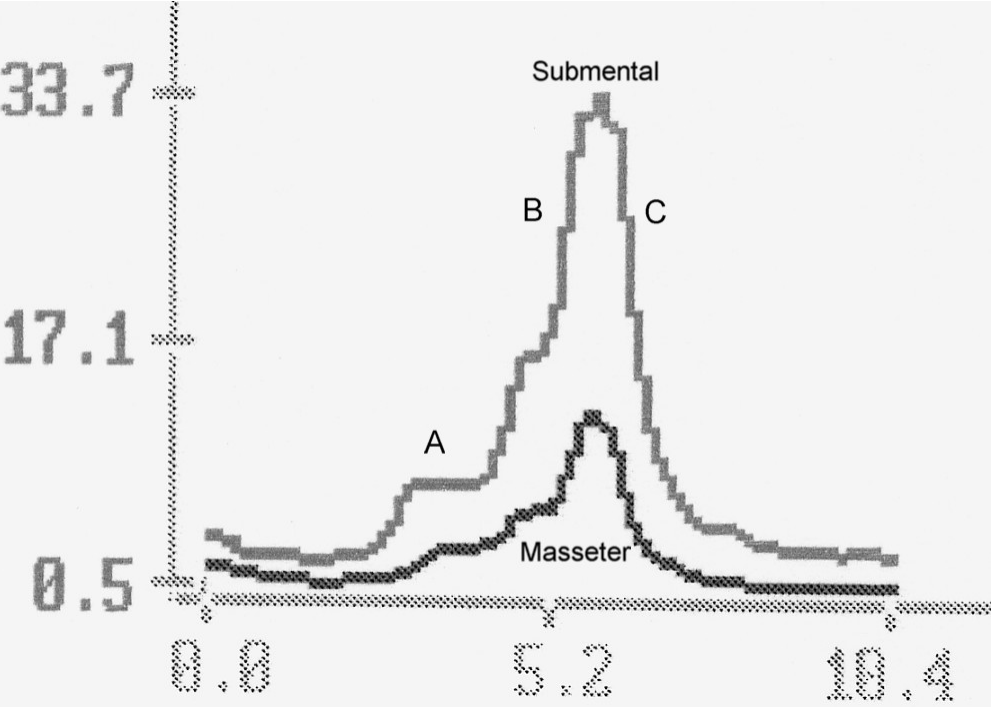
**Stages of the normal swallow (reflex part)**. A – final oral stage, B – pharyngeal stage, C – beginning of oesophageal stage. In normal deglutition the INF record is the lowest and less informative (eliminated here for clarity).

A typical single water swallow of a healthy individual is observed graphically at the rectified and low-pass filtered SEMG as a normal wave with upward deflections and a sharp apex when recorded from the MS, SUB and, to lesser extent, from INF locations [31-36]. The reflex part of a swallow is recorded from the MS, SUB and INF locations, while the conscious part can be recorded from the OO and MS locations. This upward stroke, as recorded from the MS, SUB and much less from INF locations, can be divided into three main parts (Fig. 2). In swallowing water, the first part (A) is usually seen as a mild elevation of the line and represents the final oral stage of a swallow which occurs when the tongue is moved so as to squeeze the liquid volume against the hard palate. Submental muscles and the masseter muscle support the tongue-induced pressure. At this stage, the automatic reflexive motion of swallowing is triggered. When the reflex is initiated, the second stage, the pharyngeal, begins. It is seen as a rapid voltage line elevation on the second part of the wave (B). When the bolus is finally passed into the oesophagus by the relaxation of the sphincter at the cricopharyngeal juncture, the third part (C) of the stroke is seen as a rapid descent of the SEMG line when the muscles relax and their voltage decreases. This part indicates the initiation of the oesophageal stage of swallowing. In all healthy people during the tests, the mean electric activity at SUB location is 30–50% higher than the activity at MS and INF locations. Amplitude of the OO line is usually significantly higher than the SUB and especially with the MS and INF graphic lines. Being under conscious control, the oral phase of swallowing is very variable and should be taken into consideration with caution during the evaluation of the recordings. By the same reason, the data taken from the OO electrode location is the least informative and safely can be omitted if we deal with pharyngo-laryngeal or oesophageal problems rather than with oro-maxillo-facial.

There is no visible difference between the shapes of SEMG recordings of swallows based on gender [31-36,38-44]. Elderly patients (age 70+) showed age-induced peculiarities in the recorded swallows. In general, prevalence of dysphagia increases with age and poses particular problems in the older patient, potentially compromising nutritional status, complicating the administration of solid medications, increasing the risk of aspiration pneumonia and undermining the quality of life [59]. For them, the muscle activity is usually longer in duration, and suggests a lack of coordination between activities of different muscles involved in deglutition both in single swallows and continuous drinking. For children, the duration of muscle activity during swallows and drinking in all tests showed decrease with the age, and this tendency is statistically significant. There is no statistically significant difference in electric amplitude measurements between children and adults [36].

Slow swallows and drinking are usually observed in cases of various neurological disorders affecting deglutition [60-63]. Analyzing timing one needs to remember that the times indicated by an SEMG device represent the duration of SEMG activity which lasted longer than the actual time required to pass a bolus from the oral cavity to the oesophagus.

**Figure 3:**
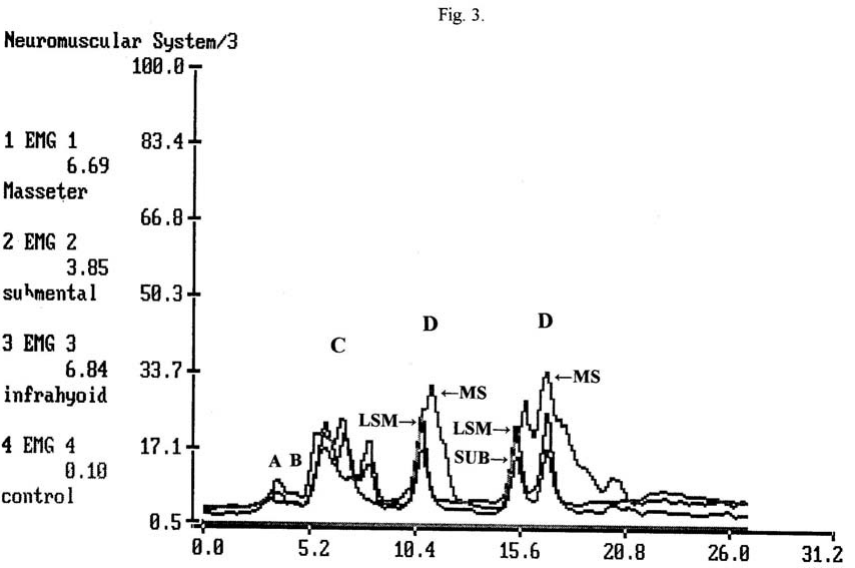
**A typical single swallow + regurgitation peak + secondary swallow of a person with Zenker's diverticulum (MS, SUB and INF locations)**. The complete swallow (2.5–4.5 period) is slightly longer than the normal swallow. The SUB peak is normal, MS peak is high and appears in front of the SUB peak, LSM is normal. After a short pause (4.5–5.5) regurgitation peaks appeared with high INF line and low MS and SUB lines (5.5–9 period). Then the secondary swallow of the regurgitated bolus followed with high SUB peak (9.5–11).

**Figure 4:**
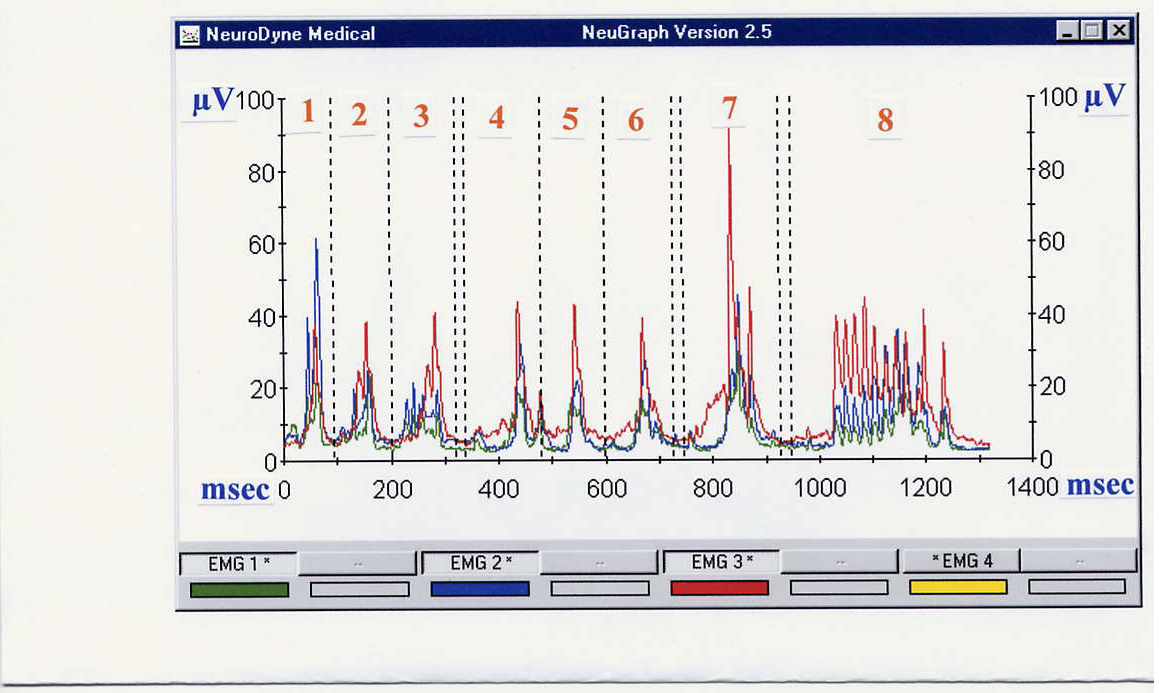
**Typical single swallows and drinking of a person with recurrent tonsillitis, age 24 years, (MS, SUB and INF locations)**. Trials 1–3 – saliva swallows, trials 4–6 – normal swallows, trial 7 – excessive swallow, trial 8 – 100 ml drinking. The SUB peaks are normal (blue line) except trial 1; MS peaks are somewhat high, especially in single swallows being almost similar to the SUB amplitude (green line), INF is very high compare to normal database (red line).

Electric amplitude is also considered in the SEMG analysis. The range (amplitude, in μV) and mean of electric activity are less important for stage-by-stage evaluation of a SEMG recording [32-34]. These data might be useful, however, when abnormal swallows are investigated. For example, a person usually presents low electric activity at the MS location after undergoing a tooth extraction [39]. Patients with the Zenker's diverticulum present unusually high electric activity at the LSM location. The main sEMG patterns of Zenker's are: A) duration of swallowing and drinking is longer than normal; B) electric amplitude of laryngeal strap muscles during swallowing activity is higher than normal; C) regurgitation peaks immediately after swallow followed by secondary swallow of the regurgitated portion of a bolus as seen at the sEMG records are specific graphic patterns (Fig. 3) [44]. Patients with recurrent tonsillitis present abnormally high electric activity of LSM and infrahyoid muscles. Acute tonsillitis and recurrent tonsillitis affect muscle activity significantly by involving additional muscles (mainly infrahyoid) in swallowing (Fig. 4). Acute tonsillitis triggers temporary electric hyperactivity of LSM and infrahyoid muscles. Recurrent tonsillitis affects MS and infrahyoid even during periods of remission (fixed pathologic changes) [40-43]. There were also numerous reports of authors who indicated changes of the masseter electric activity in patients with diseases of temporomandibular joint [64-66].

Published studies [67,68] on normal subjects show a very wide range of normal electric amplitudes for SEMG studies. These variations are not only due to biologic causes but are also greatly affected by such technical factors as skin/electrode impedance, depth of the muscle from the skin surface, location of the recording electrodes in relation to anatomic structures, variation in muscle size among individuals, and temperature. It is because of the wide variation in the normal values that an absolute value of the amplitude is considered less clinically useful.

The SEMG amplitude, however, remains an important aspect in the relationship between muscle force and the associated electric activity. There is, however, no simple relationship between an SEMG signal and muscle force. When all the different types of neuromuscular disorders are considered collectively, amplitudes are by far the most informative features [69]. Some authors argue that amplitudes are the only components that have a direct relationship to clinical symptoms (muscle weakness) in neurogenic lesions [70].

Also, during SEMG testing, there is a certain amount of impedance noise that arises directly from the resistance of the electrodes' connection to the skin. This makes skin resistance a significant factor when working with the low-level SEMG signals typical of small muscles involved in swallowing. Wiping the skin with isopropyl alcohol in a water solution has proven to be the best form of preparation for most situations. The alcohol removes the dead skin and surface oils, and the water moistens the skin and provides improved ion flow. The SEMG sensors we used are designed so that the use of electrode gel is generally not necessary.

Combined analysis of timing, amplitude and a shape of the graphic record during the screening procedure is presented in the Table 3. The data for the Table is collected from numerous studies and compiled [23,28,29,38-44,57,60,61,64].

**Table 3:** Quick reference simplified set of SEMG data for screening purposes.

**Disorder**		**SEMG locations**		
	MS	SUB	INF	Additional peculiarity

	T A S	T A S	T A S	

**Neurological**	↑ ↓ Abn	↑ ↓ Abn	↑ N N	Disorganized Peaks*

**Ent**	N N N	N ↓ Abn	N ↑ N	Multiple Peaks**

Maxillofacial (Oral)***	↑ ↓ Abn	↑ N N	↑ N N	

**Gastroenterological**	N N N	↑ N Abn	↑ ↑	Abn Regurgitation Peaks

Psychogenic****	N N N	N N N	N N N	Shoulder Tension

Abbreviations: MS – masseter electrode location, SUB – submental electrode location, INF – infrahyoid electrode location; T – timing, A – electric amplitude, S – shape of a graphic record; N – normal, AbN – abnormal, ↑ – higher than normal, ↓ – lower than normal.

* Appearance of MS, SUB and INF peaks is not coordinated

** One bolus can be swallowed in several shares

*** Including salivary gland diseases

**** Excluding malingering

This study emphasizes that a surface SEMG analysis of all the muscle groups involved in swallowing process, following a proper placement of the electrodes and selection of tests, can give reliable indications of muscle activity and provide data for screening evaluation of complaints a patient came with. Further investigation might help to develop a proper combination of flexible endoscopic evaluation of swallowing (FEES) with a nasopharyngoscope (or flexible endoscopic evaluation of swallowing with sensory testing, FEESST)[71] with SEMG to achieve complete evaluation of swallowing without exposure to radiation. Such screening might help a general practitioner to direct a patient to a neurologist, ENT physician or other proper specialist and to a specific radiographic or, perhaps, manofluorographic investigation [72-74]. Other topics for further investigation might be SEMG detecting of sensory predeglutitive oropharyngeal swallowing disorders, detecting dysphagia in patients after head and neck tumor therapy.

Dysphagia due to neurologic problems, however, might present difficulties when investigated by surface EMG. It may be possible to collect data using surface electrodes, sufficient to make conclusions with respect to patients with odynophagia or non-neurological disorders such as ENT disturbances. It is, however, difficult to nearly impossible to collect proper knowledge about neurogenic dysphagia including important and urgent etiologies such as stroke, ALS and so on. While surface kind of recording picks up not only muscle activity related with swallowing but also that of some other contiguous muscles with random or associated activity during swallowing, direct and precise neurologic assessment seems problematic. Despite that, screening assessment might be successful even in neurological cases if the record shows abnormal timing (prolongation), abnormal voltage (decreased) and abnormal shape of a signal (no peak).

In this respect it might be added that neddle EMG provides valuable data when neurologic problems are investigated. Numerous researches using needle EMG contributed significantly to our knowledge of single muscle actions during deglutition [75,76]. Specifically these studies were important for neurologic diseases [77]. Technically, however, needle EMG is an invasive and time-consuming investigation requiring expensive devices and being potentially dangerous in the neck area [76]. While this type of EMG testing might be invaluable to establish precise diagnosis, this method might be hardly recommended as a screening technique.

**Figure 5:**
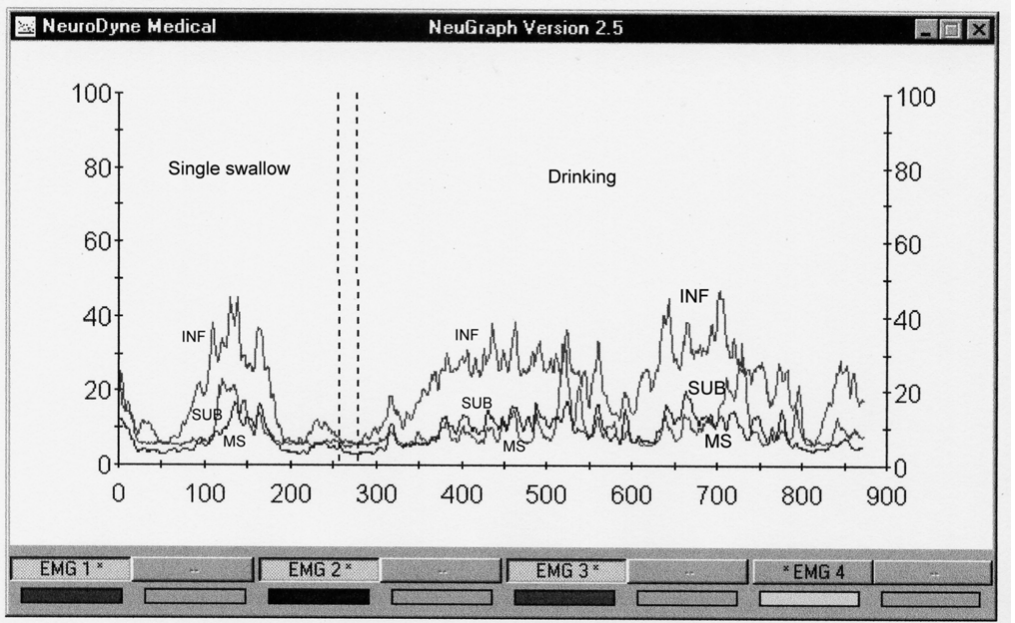
**An example of a single swallow and normal drinking of 100 cc of water by a patient with severe throat problem (dysphagia and odynophagia due to tonsillectomy, second postoperative day, pain score 7)**. MS – masseter activity, SUB – submental activity, INF – infrahyoid activity. (In real EMG records these lines are of different colors). INF line is very high (normally this line is the lowest at the record), SUB line is lower than normal and almost similar to MS line. The single swallow is prolonged and done in two shares. Drinking is arrhythmic, swallows are small.

Surface EMG assessment of odynophagia also requires special attention. Pain is a subjective experience and one may argue that it is impossible to be assessed objectively. Partially this is correct. However, since Johann Bohn's (1686) observations of reflex movements in decapitated frogs, medical scientists are aware about muscular reactions on irritation of pain receptors. While pain is subjective in general, these muscle reactions are objective and EMG can provide us with qualitative and quantitative data for their assessment (Fig. 5) [39,42]. In fact, since electricity was introduced for diagnostic purposes by Duchenne de Boulogne in 1850s–70s, these muscular reactions to painful stimulation being studied before for neurophysiological purposes, gained additional clinical importance [78]. During this period of time and up to 1930s, however, Faradic current was used for muscle testing instead of non-irritating modern EMG [79]. It helped, for example, to improve the differential diagnosis between central and peripheral paralyses and attempts were made to investigate dysphagia after stroke [80]. But naturally, no screening methods were proposed, physicians' interest shifted to electrotherapy and further EMG investigation of dysphagia/odynophagia was delayed.

It is estimated that 15 million patients suffered from a swallowing disorder during each year in the United States alone [81,82]. These data make us to believe that the screening method for this disorder might be a very timely addition to our evaluation techniques.

## Summary

Surface SEMG of swallowing is a simple and reliable method for screening and initial evaluation of dysphagia and odynophagia complaints of various origins. This noninvasive radiation-free examination has low level of discomfort, simple, time-saving and inexpensive. With proper standard technique and established normative database surface SEMG can serve as a reliable screening method for assessment of dysphasia of unknown origin in order to refer a patient to a neurologist or some other proper specialist and proper further investigation. The findings are the impetus for further study regarding the mechanisms of muscle activity changes in disorders affecting swallowing.

## Consent

Written informed consent was obtained from the patient for publication of accompanying image. A copy of the written consent is available for review by the Editor-in-Chief of this journal.

## Competing interests

The authors declare that they have no competing interests.

## Authors' contributions

General concept, data collection, assessment, writing.
